# Unraveling the Complexity of Atypical Serological Profiles in Chronic Hepatitis B: Insights Into Disease Dynamics and Clinical Implications

**DOI:** 10.7759/cureus.44899

**Published:** 2023-09-08

**Authors:** Orçun Barkay, Serpil Erol, Seniha Senbayrak

**Affiliations:** 1 Infectious Diseases and Clinical Microbiology, Faculty of Medicine, Erzincan Binali Yildirim University, Erzincan, TUR; 2 Infectious Diseases, Health Sciences University Haydarpaşa Numune Research and Training Hospital, Istanbul, TUR

**Keywords:** chronic hepatitis b (chb), anti-hbe, hbeag, anti-hbc, anti-hbs, hbsag, hbv-dna, hbv, atypical serological markers

## Abstract

Introduction

Chronic hepatitis B (CHB) continues to be a significant global public health problem. Conventional serological markers play a pivotal role in diagnosing and prognosticating CHB, but atypical serological profiles deviating from established norms pose challenges.

Methods

A cohort of 35 CHB patients who did not receive an antiviral treatment with atypical serological markers was followed for five years (2017-2022). Demographics, serological parameters, and changes were documented. Serological parameters and serum viral loads (hepatitis B virus (HBV)-deoxyribonucleic acid (DNA) levels) were assayed at the central laboratory during their routine follow-ups. Three groups of atypical serological markers are defined: hepatitis B surface antigen (HBsAg) and hepatitis B surface antibody (anti-HBs) positivity; hepatitis B e antigen (HBeAg) and anti-hepatitis B e-antigen (anti-HBe) positivity; and isolated core (anti-hepatitis B core (anti-HBc) immunoglobulin G (IgG)) positivity. Patients with concomitant HBsAg and anti-HBs were also stratified into seroreversion groups. Changes in serological markers and HBV-DNA levels across the study period were documented and evaluated at the end of the study period. Statistical analysis was conducted using the Kruskal-Wallis test and IBM SPSS Statistics software for Windows, Version 23.0 (IBM Corp., Armonk, NY, USA).

Results

In a cohort of 35 patients with atypical hepatitis B serology, demographic analysis revealed that 51.4% (n=18) were female and 48.6% (n=17) were male, with a mean age of 45.7 years. Educational distribution showed that 45.7% (n=16) completed primary education, 22.8% (n=8) had a high school education, and 31.5% (n=11) held university degrees. Among these patients, 10 displayed the concurrent presence of HBsAg and anti-HBs, with 60% (n=6) being female. Serum HBV-DNA was detectable in all cases. After five years, 60% (n=6) exhibited seroconversion from HBsAg to anti-HBs, particularly notable in females (66.7%). These patients showed lower HBsAg titers and serum HBV-DNA levels (p = 0.048, p = 0.036). A subset of 15 patients demonstrated simultaneous HBeAg and anti-HBe positivity. The HBeAg seropositivity waned over time, with 40% (n=6) and 26.7% (n=4) females and males, respectively, retaining positivity by the fifth year. During this period, serum HBV-DNA levels decreased. The remaining five patients sustained HBeAg and anti-HBe positivity. Among 10 patients solely positive for anti-HBc IgG, three had concurrent HBV-DNA positivity. Strikingly, three patients with negative HBV-DNA developed anti-HBs positivity after five years.

Conclusion

The complexity of CHB infection demands a comprehensive understanding. Atypical serological profiles suggest distinct disease stages, immune response variations, and viral mutations. This study enhances comprehension of viral replication, immune responses, and disease progression, potentially guiding tailored therapeutic strategies.

## Introduction

Chronic hepatitis B (CHB) infection, caused by the hepatitis B virus (HBV), remains a substantial global health challenge with potentially severe outcomes such as cirrhosis and hepatocellular carcinoma [1]. The conventional serological markers, including hepatitis B surface antigen (HBsAg), hepatitis B e antigen (HBeAg), and hepatitis B core antibody (anti-HBc), have played a pivotal role in diagnosing and prognosticating CHB [2,3]. However, within this context, a subset of patients present with atypical serological profiles that deviate from the established norms. These profiles encompass a phenomenon such as the simultaneous presence of HBsAg and hepatitis B surface antibodies (anti-HBs), as well as the dynamic interplay between HBeAg and anti-HBe, revealing the intricate and nuanced nature of CHB infection [4,5].

Exploring these atypical serological markers is crucial to gaining a comprehensive understanding of the virological and immunological underpinnings of CHB. Such deviations have prompted scientific interest as they potentially signify distinct stages of the disease, variations in immune response, or even the emergence of viral mutations. As we delve into this uncharted territory, it becomes evident that these deviations hold the potential to provide novel insights into disease progression, treatment response, and the overall clinical management of CHB patients.

This study aspires to contribute novel insights into the realm of atypical serological profiles in CHB patients. Our findings have the potential to enhance our understanding of the intricate interplay between viral replication, immune response dynamics, and disease progression. Moreover, the knowledge gained from this investigation may guide the development of personalized prognostic markers and tailored therapeutic strategies for patients presenting with these unique serological variations.

## Materials and methods

Patient enrollment and demographic data

Hepatitis B surface antigen positivity for at least six months is being considered CHB. These CHB patients are routinely followed up at our clinic. Serological parameters are the most appropriate laboratory parameters used for follow-up. Sometimes unexpected results from these parameters can be detected. We used a cohort comprising 35 individuals diagnosed with CHB infection and exhibiting unconventional serological markers. These markers were prospectively assembled from patients under routine follow-up at our clinical facility. Exclusion criteria encompassed individuals receiving antiviral treatment. The observational study spanned a duration of five years, commencing in January 2017 and concluding in December 2022. Vital demographic details were extracted from the hospital information system and patient follow-up records.

Serological parameter assessment

Serum samples collected from the enrolled patients were meticulously subjected to serological parameter analysis at the central laboratory. The parameters of interest included HBsAg titers, anti-HBs, HBeAg, anti-HBe, and HBV-DNA levels.

Data collection and validation

All resultant data were systematically retrieved from the hospital information system. Any initially anomalous test results were validated through replication at subsequent appointments, ensuring the reliability of the obtained data.

Patient stratification and analysis

Among the cohort, patients presenting simultaneous positivity for both HBsAg and anti-HBs were evaluated and stratified into two distinct groups for further investigation. The first group consisted of patients who subsequently manifested anti-HBs development, while the second group comprised those in whom this development did not occur. The statistical correlation between mean serum HBsAg titer and HBV-DNA levels within these groups was scrutinized using the Kruskal-Wallis test, with statistical significance denoted by a p-value of ≤ 0.05. Patients showing HBeAg and anti-HBe positivity and patients having isolated anti-HBc Ig G positivity are also evaluated. IBM SPSS Statistics for Windows, Version 23.0 (IBM Corp., Armonk, NY) facilitated the statistical analysis.

Ethical considerations

Approval from the local ethics committee of the Clinical Research Ethics Committee of the Erzincan Binali Yıldırım University, Erzincan, Turkey (Date: March 3, 2023/Decision No. 07/3) was secured for the study. Informed consent was duly obtained from each participating patient.

## Results

Among the cohort of 35 patients exhibiting atypical hepatitis B serology, 18 (51.4%) were of the female gender, while 17 (48.6%) were male. The average age of the cohort was 45.7 years. Out of these patients, 16 (45.7%) had completed primary education, eight (22.8%) had attained a high school education, and 11 (31.5%) held university degrees. Comprehensive demographic information about the patients can be found in Table 1.

**Table 1:** Demographic characteristics of the patients *S.D.: standard deviation; HBsAg: hepatitis B surface antigen; anti-HBs: hepatitis B surface antibody; HBeAg: hepatitis B e antigen; anti-HBe: anti-hepatitis B e-antigen; anti-HBc: anti-hepatitis B core; IgG: immunoglobulin G

Demographic Parameters	Groups of Atypical Serological Markers			Total
	HBsAg and Anti-HBs Positivity	HBeAg and Anti-HBe Positivity	Anti-HBc IgG Positivity	
Mean Age (In Years) (S.D.*)	44.6 ( ± 9,04)	46.3 ( ± 9,83)	46.2 ( ± 4,91)	45.7 ( ± 8,27)
Gender (Male/Female)				
	4	5	8	17
	6	10	2	18
Education Status (Elementary/ High School/ University)				
	5	9	2	16
	3	2	3	8
	2	4	5	11

Among the cohort, 10 patients displayed the concomitant presence of HBsAg and anti-HBs. Of these patients, six (60%) were female and four (40%) were male. The mean age of this group was 44.6 years. Serum hepatitis B virus (HBV)-deoxyribonucleic acid (DNA) levels were detectable in all cases. After a five-year interval, six (60%) of these patients exhibited seroconversion with negativity for HBsAg and the emergence of anti-HBs. Of these patients, two (33.3%) were male and four (66.7%) were female. Notably, these patients displayed lower mean HBsAg titers and serum HBV-DNA levels compared to the remaining group, and this distinction held statistical significance (p = 0.048, p = 0.036) (Table 2).

**Table 2:** Mean HBsAg titers and serum HBV-DNA levels of anti-HBs developers and non-developers *Kruskal-Wallis Test anti-HBs: hepatitis B surface antibody; HBsAg: hepatitis B surface antigen; HBV: hepatitis B virus; DNA: deoxyribonucleic acid

Laboratory Parameters	Anti-HBs Developer	N	Mean	SD	Percentiles			P*
					25th	Median	75th	
HBsAg Titer	Yes	6	3472.69	3612.075	825.00	1710.00	3680.00	0.048
	No	4	4146.58	4276.279	1013.00	2276.00	4458.00	
HBV-DNA Level (IU/L)	Yes	6	670	600.50	128.50	264.00	520.00	0.036
	No	4	2015	2050.50	580.00	1180.00	2110.00	

A subset of 15 patients displayed simultaneous HBeAg and anti-HBe positivity, and in this group, there were 10 (66.7%) females and five (33.3%) males. The HBeAg seropositivity waned in five patients during the third year, with this number rising to 10, including six (40%) females and four (26.7%) males by the fifth year. During this period, serum HBV-DNA levels demonstrated a decline. The remaining five patients sustained HBeAg and anti-HBe positivity. Of the 10 patients solely positive for anti-HBc IgG, three displayed concurrent HBV-DNA positivity. Intriguingly, three patients with negative HBV-DNA levels developed anti-HBs positivity after a five-year interval. All patients are still being followed.

## Discussion

The findings of our study provide a comprehensive understanding of the intricate dynamics within the atypical serological profiles observed in CHB patients. By correlating our results with existing literature, we can glean deeper insights into the potential clinical implications of these deviations. Our study findings also hold valuable insights that contribute to the accumulation of knowledge crucial for the global management and control of this disease burden [6]. As we strive to understand the intricate complexities of atypical serological profiles in CHB patients, our research advances broader efforts to alleviate the impact of this disease on a global scale.

In the context of the known natural history of CHB, the coexistence of HBsAg and anti-HBs in certain patients challenges conventional interpretations [1,7,8]. This phenomenon may indicate transitions between distinct phases of infection, potentially involving dynamic shifts in viral replication and immune response [9]. Aligning with the concept of complex phases proposed by Croagh and Lubel, our study underscores the importance of nuanced categorizations to accurately depict the disease trajectory [10].

Moreover, the observation of seroreversion highlights the delicate balance between viral replication and immune control [11]. The direct correlation between serum HBsAg titers and HBV-DNA levels among patients undergoing seroreversion echoes the notion of viral replication influencing serological markers [5]. This dynamic interaction resonates with the immune modulation phases postulated by Chisari and Ferrari, suggesting shifts between immune containment and viral replication [12].

The subset of patients displaying simultaneous HBeAg and anti-HBe positivity introduces another layer of complexity [13]. This phenomenon could signify an evolving balance between viral replication and immune responses [14]. The temporal dynamics of HBeAg seropositivity, as observed in our study, may represent transitions through distinct stages of immune control, mirroring the dynamic immune phases proposed by Chisari and Ferrari [12]. These nuanced observations warrant further exploration to delineate the underlying mechanisms and potential prognostic implications.

The emergence of anti-HBs positivity in patients initially positive for anti-HBc IgG signifies a shift in the immune landscape [5]. This intriguing finding aligns with Rehermann et al.'s concept of prolonged immune responses even after acute viral hepatitis recovery [15]. The factors triggering this shift deserve meticulous investigation, as they could provide insights into the interplay between viral clearance and immune activation.

Limitations of the study

However, it's essential to acknowledge the limitations of our study, such as the relatively small cohort size and being held by a single center. Collaborative endeavors involving larger cohorts and extended follow-up periods are essential to validate and expand upon our insights, ultimately shaping the landscape of CHB management strategies.

## Conclusions

The investigation into atypical serological profiles in CHB patients elucidates the nuanced landscape of disease dynamics, immune responses, and viral replication. These findings not only expand our comprehension of CHB but also offer the potential to revolutionize prognostic strategies and therapeutic approaches. The unraveling of such intricacies underscores the importance of personalized medicine in CHB management and calls for continued research into the underlying mechanisms governing these atypical serological profiles.
